# Supplementary material for: A viral effector blocks the turnover of a plant NLR receptor to trigger a robust immune response
Source: EMBO J. 2024 Jul 17;43(17):7. doi: 10.1038/s44318-024-00174-6 (PMC11377725; doi:10.1038/s44318-024-00174-6)
Supplement: Supplementary file 1 — Appendix [file 44318_2024_174_MOESM1_ESM.pdf]

Table of Contents

Appendix Figure S1 Analysis of NSm or NSm^{21}-YFP's effect on the protein and transcript level of Sw-5b, SD, and NB-LRR…………………………………………………………………………………….............2

Appendix Figure S2 Structure and domain analysis of S-ribonuclease binding protein 1 (SBP1)…………..4

Appendix Figure S3. Generation and analysis of the *sbp1-1* and *sbp1-2* CRISPR KO plants……….......6

Appendix Figure S4. SBP1-KO does not alter the transcription level of *Sw-5b*, *Sw-5b^{Heinz}*, *SD*, *NB-LRR*, *R8*, and *Rpi-blb2* .. ...……………………………………………………………………………………..7

Appendix Figure S5. TRV-mediated gene silencing of E3 ligase *SlSBP1* in tomato plant carrying *Sw-5b* resistance gene…………………………………………………………………………………….......8

Appendix Figure S6. Analysis of TSWV replicon accumulation in WT, *sbp1-1* and *sbp1-2* mutant *N. benthamiana* plant leaves……………………………………………………………………………...9

Appendix Figure S7. Role of SD, and the NSm recognition region of Sw-5b LRR in the regulation of inactive and active Sw-5b protein…………………………………………………………………………10

Appendix Figure S8. Protein turnover rate of the active state vs the inactive state of GST-Sw-5b *in vitro*...11

Appendix Figure S9. Amino acid sequence alignment of Sw-5b^{Heinz}, Sw-5b and R8………………………13

Appendix Table S1. List of primers used in this study……………………………………………………14

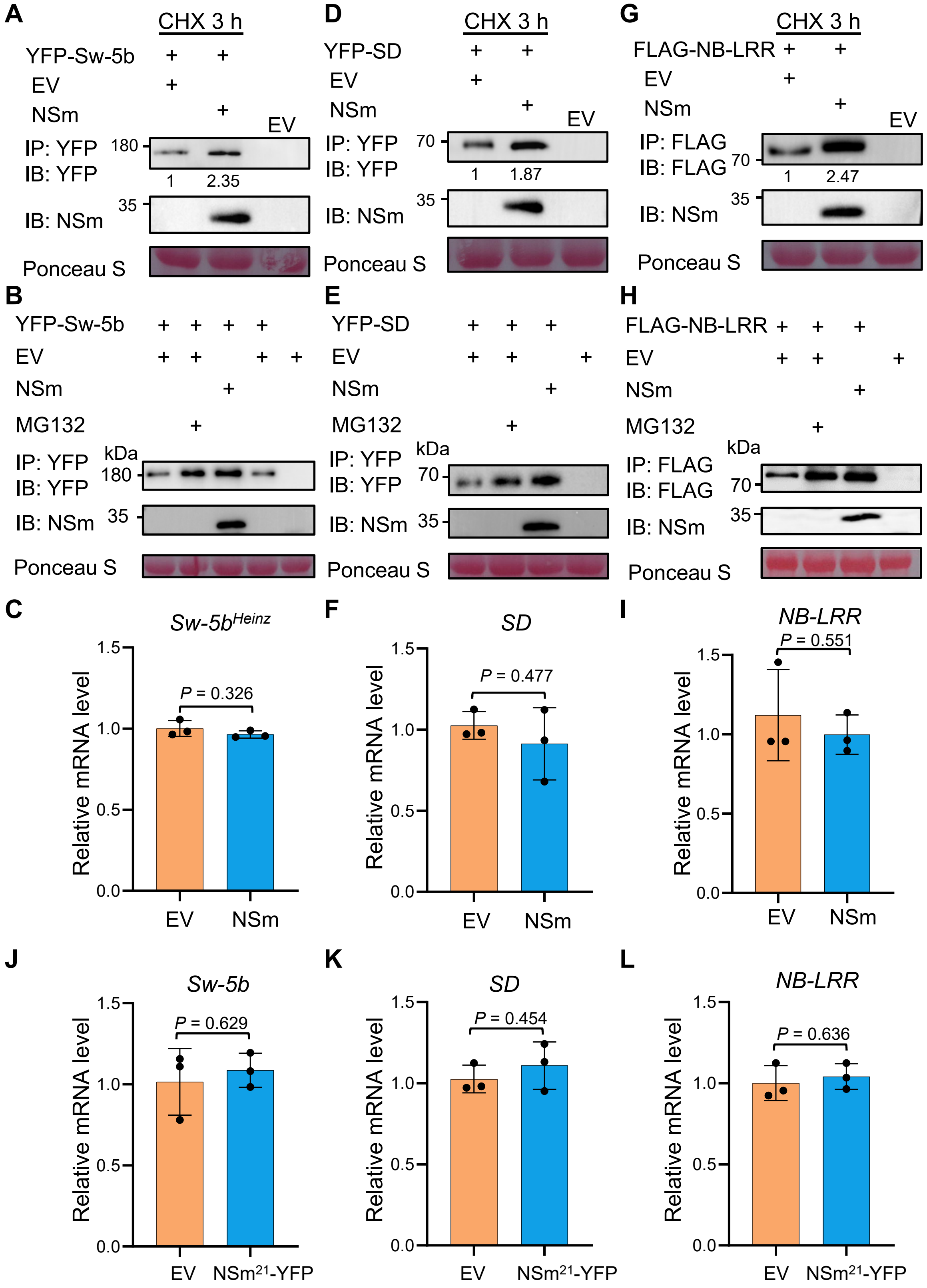

Appendix Figure S1. Analysis of TSWV NSm or NSm^{21}-YFP's effect on the protein and transcript level of Sw-5b, SD, and NB-LRR.

(A, D and G) Protein accumulation of Sw-5b, SD and NB-LRR in the absence or presence of NSm in *N. benthamiana* leaves. YFP-Sw-5b (A), YFP-SD (D) and

FLAG-NB-LRR (G) were co-expressed with the EV in one half leaf of *N. benthamiana* plants and co-expressed with NSm in another half of the same leaf and the leaves treated with 10 µg/mL CHX at 19 hours post infiltration (hpi). Samples were collected for immunoblotting at 22 hpi. (B, E and H) Protein accumulation of Sw-5b (B), SD (E) and NB-LRR (H) without or with MG132 or NSm in *N. benthamiana* leaves. 25 µM MG132 was infiltrated in plant leaves expressing YFP-Sw-5b, YFP-SD and FLAG-NB-LRR at 13 hpi and samples were collected for immunoblotting at 21 dpi. YFP-Sw-5b, YFP-SD, and FLAG-NB-LRR was co-expressed with either the EV or the NSm in *N. benthamiana* leaves, and protein accumulation was detected by immunoblot at 21 hpi using YFP, FLAG and NSm specific antibodies. For all panels: IB, immunoblot with specific antibody; IP, immunoprecipitation with specific antibody. A ponceau S stained band is shown to serve as the loading control. Protein accumulation level was quantified by ImageJ. (C, F and I) Relative RNA expression level of YFP-Sw-5b^{Heinz} (C), YFP-SD (F), and FLAG-NB-LRR (I) without or with NSm in *N. benthamiana* leaves. YFP-Sw-5b, YFP-SD, and FLAG-NB-LRR was co-expressed with EV or NSm and their RNA expression was analyzed by qRT-PCR at 21 hpi. Error bars represent SD (n = 3). (J-L) Quantification of RNA expression level of YFP-Sw-5b (J), YFP-SD (K) and FLAG-NB-LRR (L) without or with NSm^{21}-YFP in *N. benthamiana* leaves. YFP-Sw-5b, YFP-SD, and FLAG-NB-LRR were co-expressed with YFP or NSm^{21}-YFP and their RNA expression was analyzed by qRT-PCR at 21 hpi. NSm refers to the elicitor TSWV NSm. In (C), (F), (I), and (J-L), data are presented as means ± SD (n = 3 biologically independent samples). *P* values are indicated in the graphs (two-sided Student's *t*-tests). Source data are provided as a Source Data file. All experiments were repeated at least three times with similar results.

A

Number and frequency of candidates selected in Y2H screening by SD

Name	Genes	Frequency
SD interacting protein 1	*S-ribonuclease binding protein1 (SBP1)*	4
SD interacting protein 2	*Cytochrome b-c1 complex subunit 7*	2
SD interacting protein 3	*Heat stress transcription factor B*	1
⋮	⋮	⋮
SD interacting protein 26	*Heat shock protein 3*	1

Number and frequency of candidates selected in Y2H screening by NB

Name	Genes	Frequency
NB interacting protein 1	*S-ribonuclease binding protein1 (SBP1)*	2
NB interacting protein 2	*SGT1 homolog protein*	2
NB interacting protein 3	*Heat shock protein*	1
⋮	⋮	⋮
NB interacting protein 35	*Protein phosphatase 4*	3

B

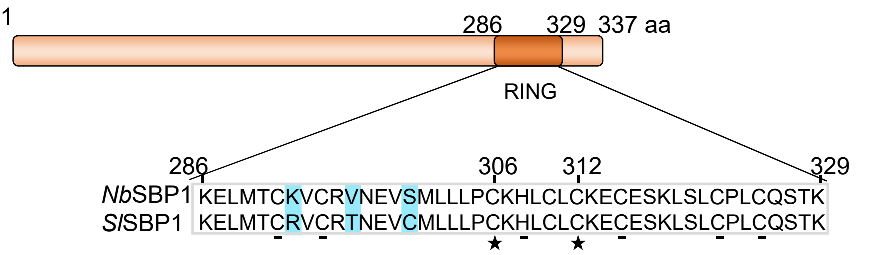

C

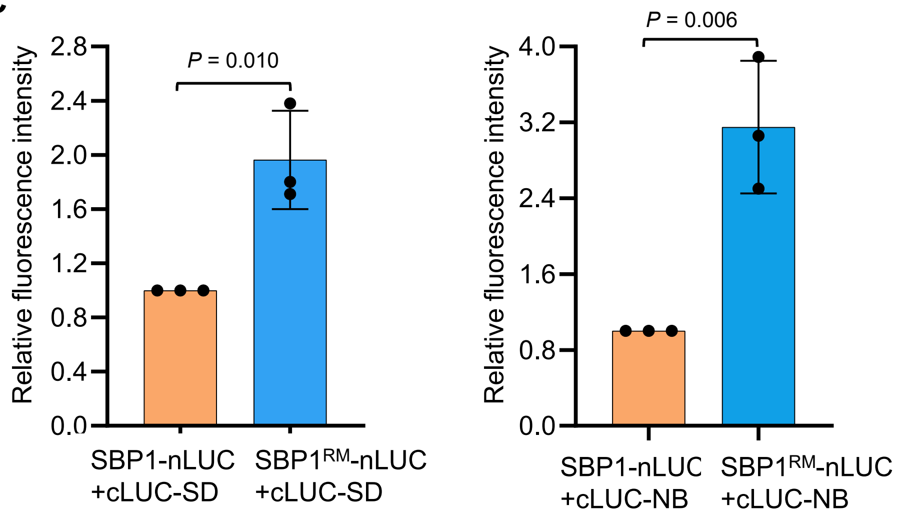

Appendix Figure S2. Structure and domain analysis of S-ribonuclease binding protein 1 (SBP1).

(A) Number of SBP1 and other candidates identified in Y2H library screening using Sw-5b SD (upper panel) or NB (lower panel) as bait. (B) Domain organization of the E3 ligase SBP1 and sequence alignment of RING domain region of *Nb*SBP1 from *N. benthamiana* and *Sl*SBP1 from *S. lycopersicum*. Key: RING, E3 catalytic domain. Cysteine and histidine residues that coordinate zinc are underlined and Cysteine residues for generating $SBP1^{RM}$ mutation are marked with star. (C) Quantification of fluorescence intensity of the interaction of SD and NB with SBP1 or $SBP1^{RM}$ from Figure 2B. Fluorescence intensity was quantified by ImageJ. Data are presented as means ± SD (n = 3 biologically independent samples). *P* values are indicated in the graphs (two-sided Student's *t*-tests). Source data are provided as a Source Data file. Experiment was repeated at least three times with similar results.

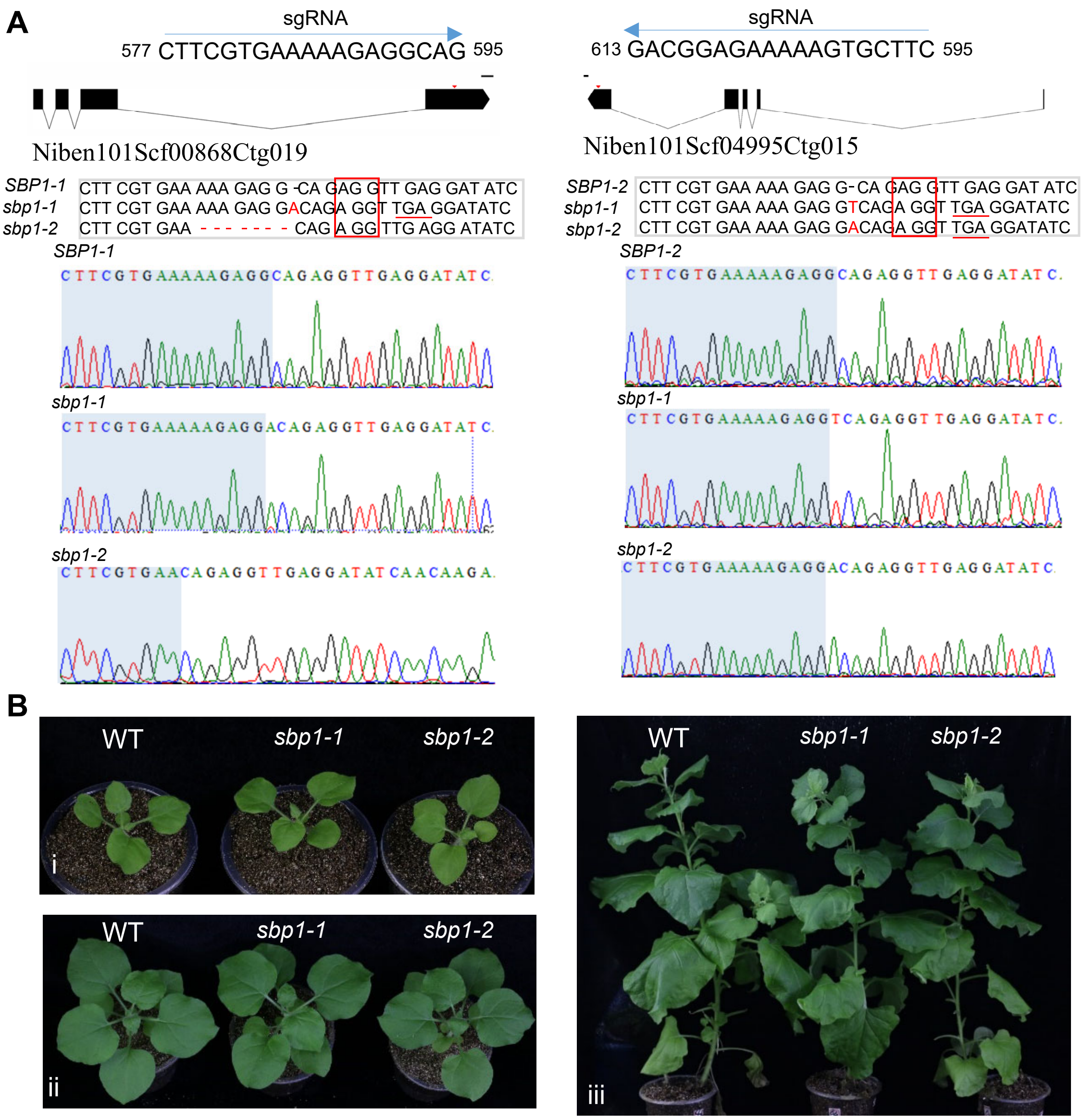

Appendix Figure S3. Generation and analysis of the *sbp1-1* and *sbp1-2* CRISPR KO plants.

(A) Generation of the *sbp1-1* and *sbp1-2* mutant lines by CRISPR/Cas9 in *N. benthamiana*. There are two close homologs of *SBP1*, *SBP1-1* (Niben101Scf00868Ctg019) and *SBP1-2* (Niben101Scf04995Ctg015) in the genome sequence of *N. benthamiana* plant. SBP1-1 and SBP1-2 has 97.44% nucleotide sequence identity. *sbp1-1* knockout line has a single nucleotide insertion upstream of the PAM NGG site (boxed region) in both *SBP1-1* and *SBP1-2*, which both lead to a frame shift mutation and a premature stop codon (underlined). *sbp1-2* has 7 nucleotide deletion upstream of the PAM NGG site in *SBP1-1* and a single nucleotide insertion upstream of the PAM NGG site in *SBP1-2*, which both lead to frame shift mutations. (B) Phenotype of *sbp1-1* and *sbp1-2* mutant lines in comparison with WT plant at 4 (i), 6 (ii), and 18 (iii) weeks post sowing.

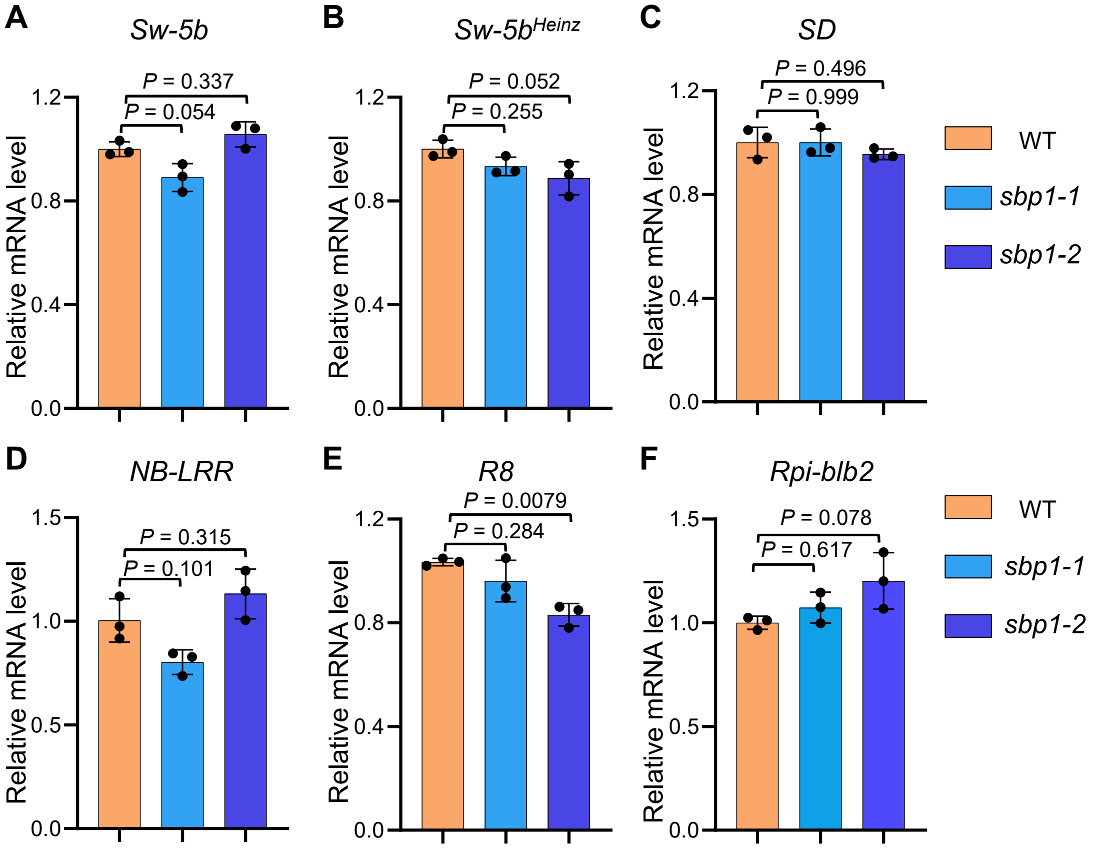

Appendix Figure S4 SBP1-KO does not alter the transcription level of *Sw-5b*, *Sw-5b*^{Heinz}, *SD*, *NB-LRR*, *R8*, and *Rpi-blb2*.

(A-D) RNA expression level of YFP-Sw-5b (A), YFP-Sw-5b^{Heinz} (B), YFP-SD (C), and FLAG-NB-LRR (D) in WT, *sbp1-1*, and *sbp1-2* plant leaves. Total RNA were extracted at 24 hpi for qRT-PCR analysis. (E and F) RNA expression level of YFP-R8 (E), and FLAG-Rpi-blb2 (F) in WT, *sbp1-1*, and *sbp1-2* plant leaves, respectively. Total RNA were extracted at 30 hpi for qRT-PCR analysis. In (A-F), data are presented as means ± SD (n = 3 biologically independent samples). *P* values are indicated in the graphs (two-sided Student's *t*-tests). Source data are provided as a Source Data file. All experiments were repeated at least three times with similar results.

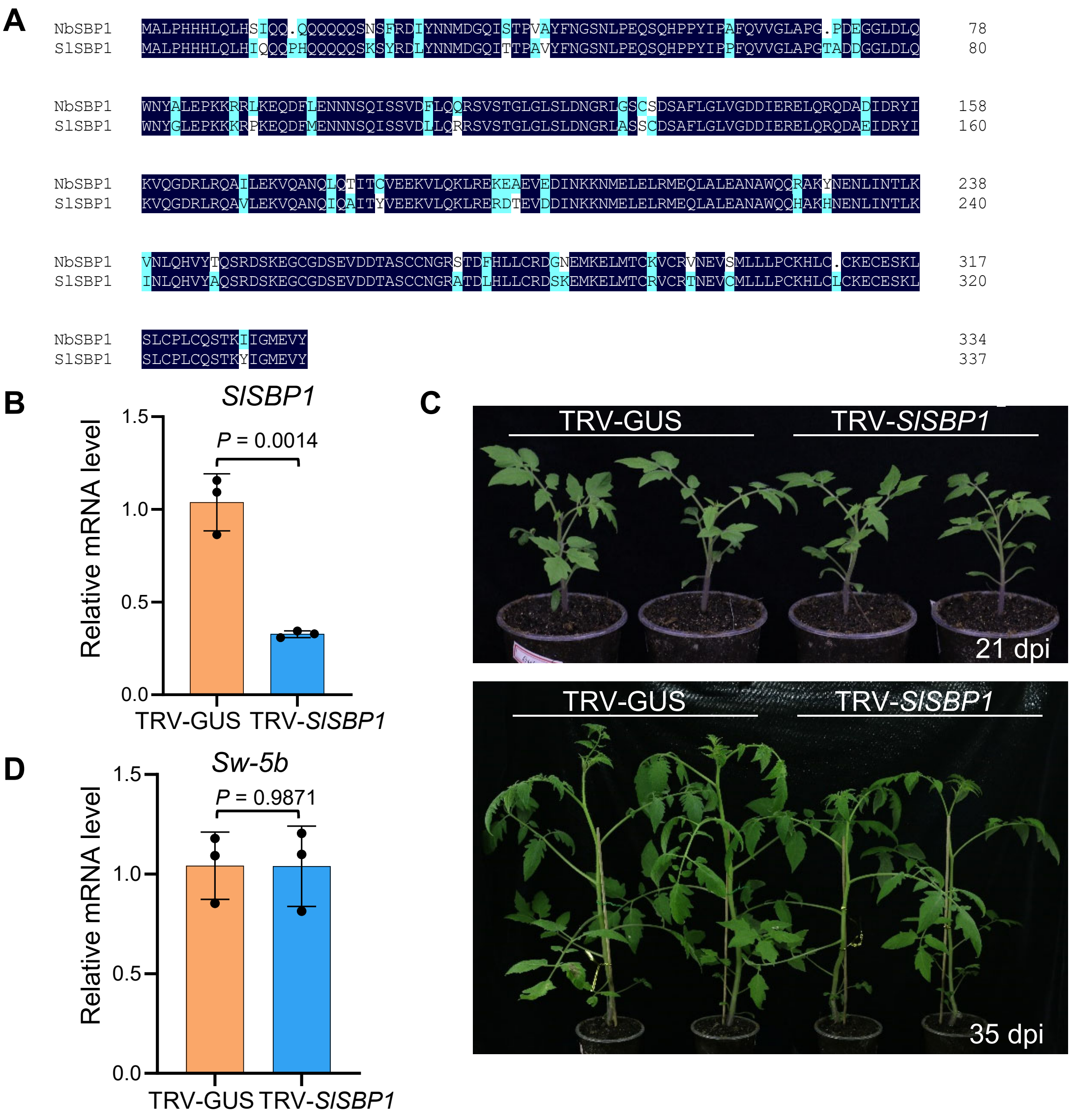

Appendix Figure S5. TRV-mediated gene silencing of E3 ligase *Sl*SBP1 in tomato plant carrying Sw-5b resistance gene.

(A) Amino acid sequence alignment of *Nb*SBP1 from *N. benthamiana* and *Sl*SBP1 from *Solanum lycopersicum. Nb*SBP1 and *Sl*SBP1 had 87.54 % amino acid sequence identity. (B) RNA expression level of *SlSBP1* in tomato cultivar 1760 carrying the Sw-5b resistance gene pretreated with TRV-*SlSBP1* or TRV-GUS. Systemic new leaves were harvested at 21 days post TRV treatment and extracted total RNA was analyzed by qRT-PCR. (C) Phenotype of TRV-*SlSBP1* and TRV-GUS treated plants. The plants were photographed at 21 days post TRV treatment (the upper panel) and at 35 days post TRV treatment (the lower panel). (D) RNA expression level of Sw-5b in tomato cultivar 1760 carrying the Sw-5b resistance gene treated with TRV-*SlSBP1* or TRV-GUS. Systemic new leaves were harvested at 35 days post TRV treatment and extracted total RNA was analyzed by qRT-PCR. In (B) and (D), data are presented as means ± SD (n = 3 biologically independent samples). *P* values are indicated in the graphs (two-sided Student's *t*-tests). Source data are provided as a Source Data file. All experiments were repeated at least three times with similar results.

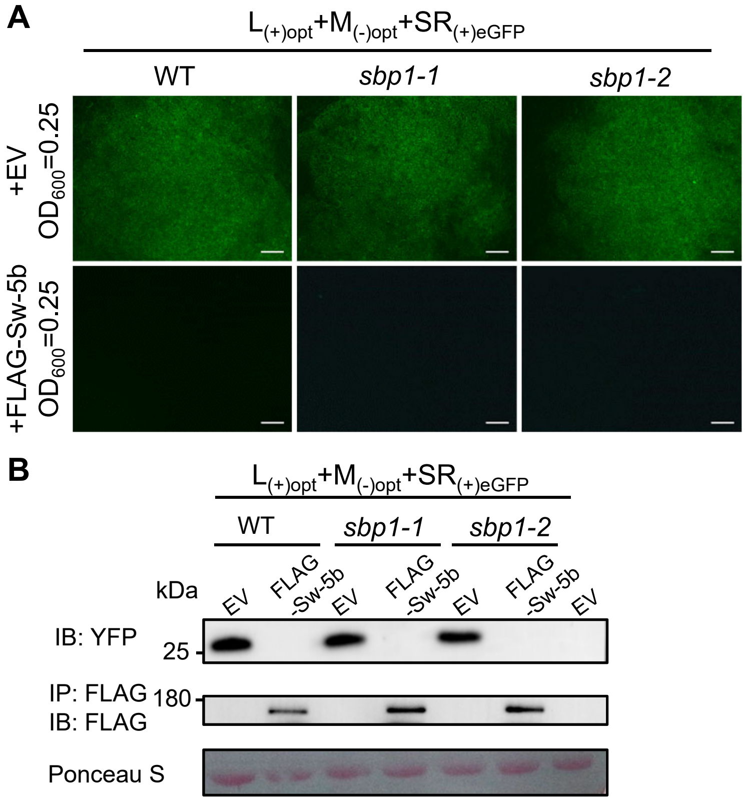

Appendix Figure S6. Analysis of TSWV replicon accumulation in WT, *sbp1-1* and *sbp1-2* mutant *N. benthamiana* plant leaves.

(A-B) The infectious clones of TSWV replicons carrying GFP reporter was co-expressed with Sw-5b (final OD600 of 0.25) or EV in WT *N. benthamiana*, *sbp1-1* and *sbp1-2* mutant plant leaves via agrobacterium. GFP fluorescence and protein accumulation were detected by inverted fluorescence microscopy (A) or Western blot using YFP specific antibodies (B) at 2 days post infiltration (dpi). The pCambia2300S vector was expressed in WT *N. benthamiana* leaves and used as a Mock control. Bar = 100 μm. The sizes of protein are shown on the left (B). Ponceau S stained band is shown to serve as the loading control. Source data are provided as a Source Data file. All experiments were repeated at least three times with similar results.

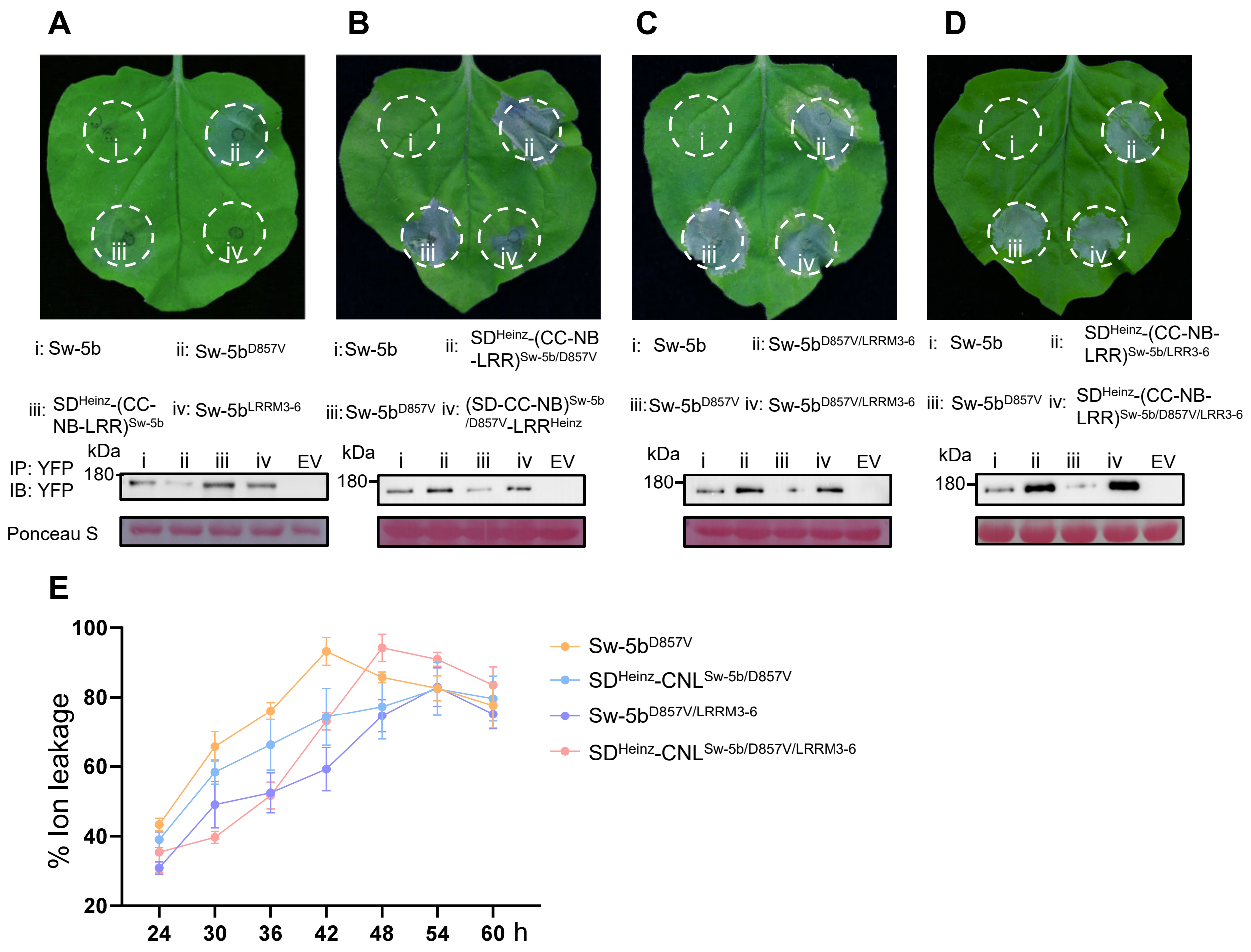

Appendix Figure S7. Role of SD, and the NSm recognition region of Sw-5b LRR in the regulation of inactive and active state Sw-5b protein.

(A-D) HR cell death and protein accumulation analysis of SD^{Heinz}-(CC-NB-LRR)$^{Sw\text{-}5b}$ and Sw-5b$^{LRRM3\text{-}6}$ (A), SD^{Heinz}-(CC-NB-LRR)$^{Sw\text{-}5b/D857V}$ and (SD-CC-NB)$^{Sw\text{-}5b/D857V}$-LRR^{Heinz} (B), Sw-5b$^{D857V/LRRM3\text{-}6}$ (C), YFP-SD^{Heinz}-(CC-NB-LRR)$^{Sw\text{-}5b/LRRM3\text{-}6}$ and YFP-SD^{Heinz}-(CC-NB-LRR)$^{Sw\text{-}5b/D857V/LRRM3\text{-}6}$ (D) mutants in *N. benthamiana* leaves. HR phenotype in the infiltrated leaves was photographed at 4 d post inoculation. Protein accumulation was detected and showed under the photos of HR. (E) Ion leakage analysis of autoactivated chimeric Sw-5b variants in *N. benthamiana* leaves at 6-hour intervals from 24 to 48 hours post agroinfiltration. Data are presented as means ± SD (n = 3 biologically independent samples). Source data are provided as a Source Data file. All experiments were repeated at least three times with similar results.

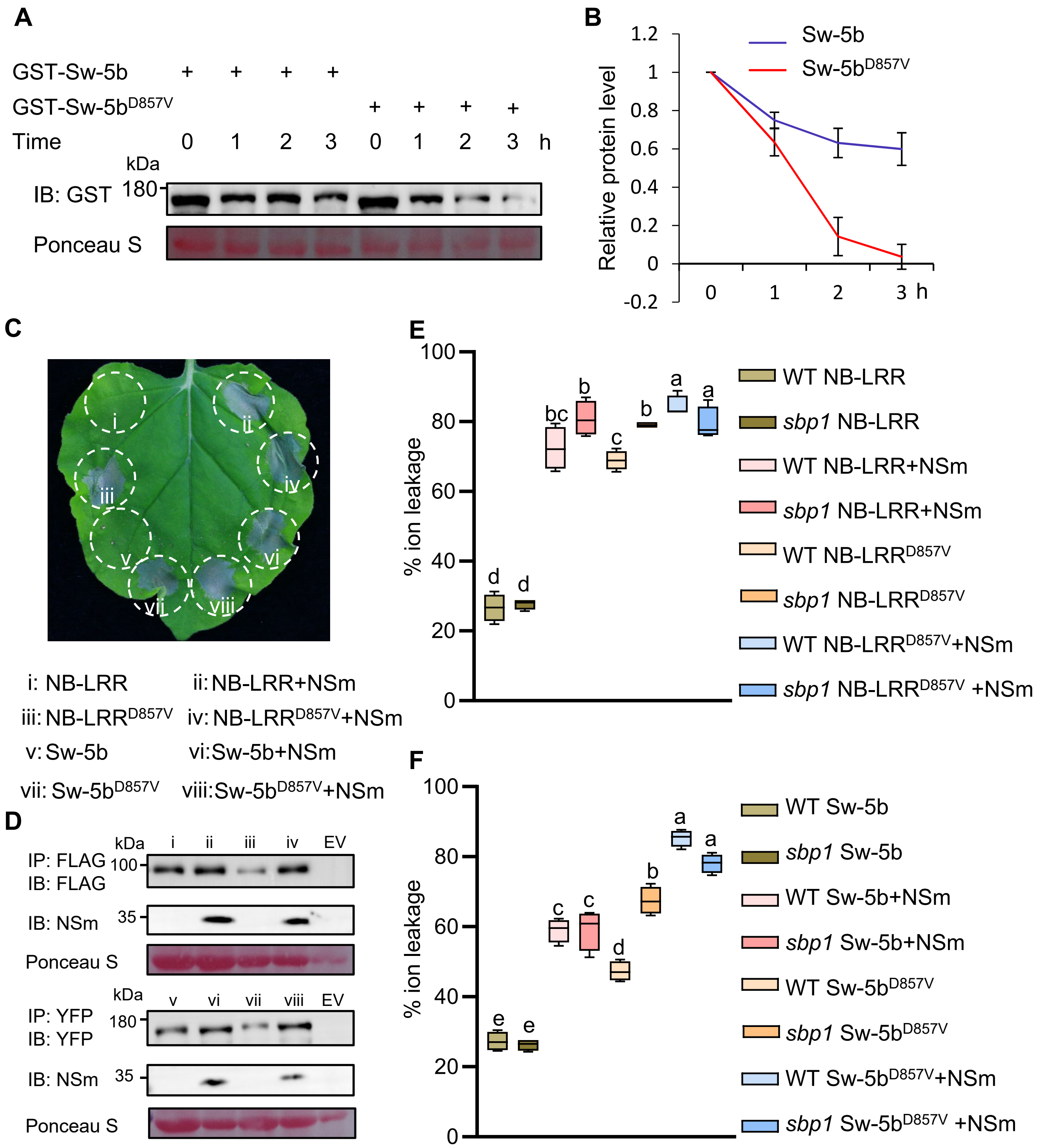

Appendix Figure S8. Protein turnover rate of the active state vs the inactive state of GST-Sw-5b *in vitro*.

(A-B) Protein turnover rate of purified GST-Sw-5b and GST-Sw-5b^{D857V} *in vitro*. GST-Sw-5b and GST-Sw-5b^{D857V} were expressed and purified from *E. coli* and mixed with total protein extracts from wild type *N. benthamiana* leaves. The mixture was incubated at room temperature for 0-3 hours. The proteins were detected by immunoblotting using GST specific antibodies. Protein accumulation level in panel (A) was quantified by ImageJ and was shown in panel (B). Data are presented as means ±

SD (n = 3 biologically independent samples). (C) HR cell death analysis of YFP-Sw-5b, YFP-Sw-5b^{D857V}, FLAG-NB-LRR and FLAG-NB-LRR^{D857V}, in the absence or the presence of TSWV NSm in WT *N. benthamiana* leaves. HR phenotype in the infiltrated leaves was photographed at 4 d post inoculation. (D) Protein accumulation in (C) was detected using FLAG, YFP, and NSm specific antibodies. (E and F) Ion leakage analysis of Sw-5b, Sw-5b^{D857V}, NB-LRR and NB-LRR^{D857V} in the absence or the presence of TSWV NSm in WT or *SBP1* knockout *N. benthamiana* leaves at 6-hour intervals from 24 to 48 hours post agroinfiltration. Data are shown as the box plots with the interquartile range as the upper and lower confines, minima and maxima as whiskers, and the median as a solid line (n = 4); the exact *P* values are shown in the Source Data (one-way ANOVA; a-e represent statistically different groups). NSm refers to the elicitor TSWV NSm. Source data are provided as a Source Data file. All experiments were repeated at least three times with similar results.

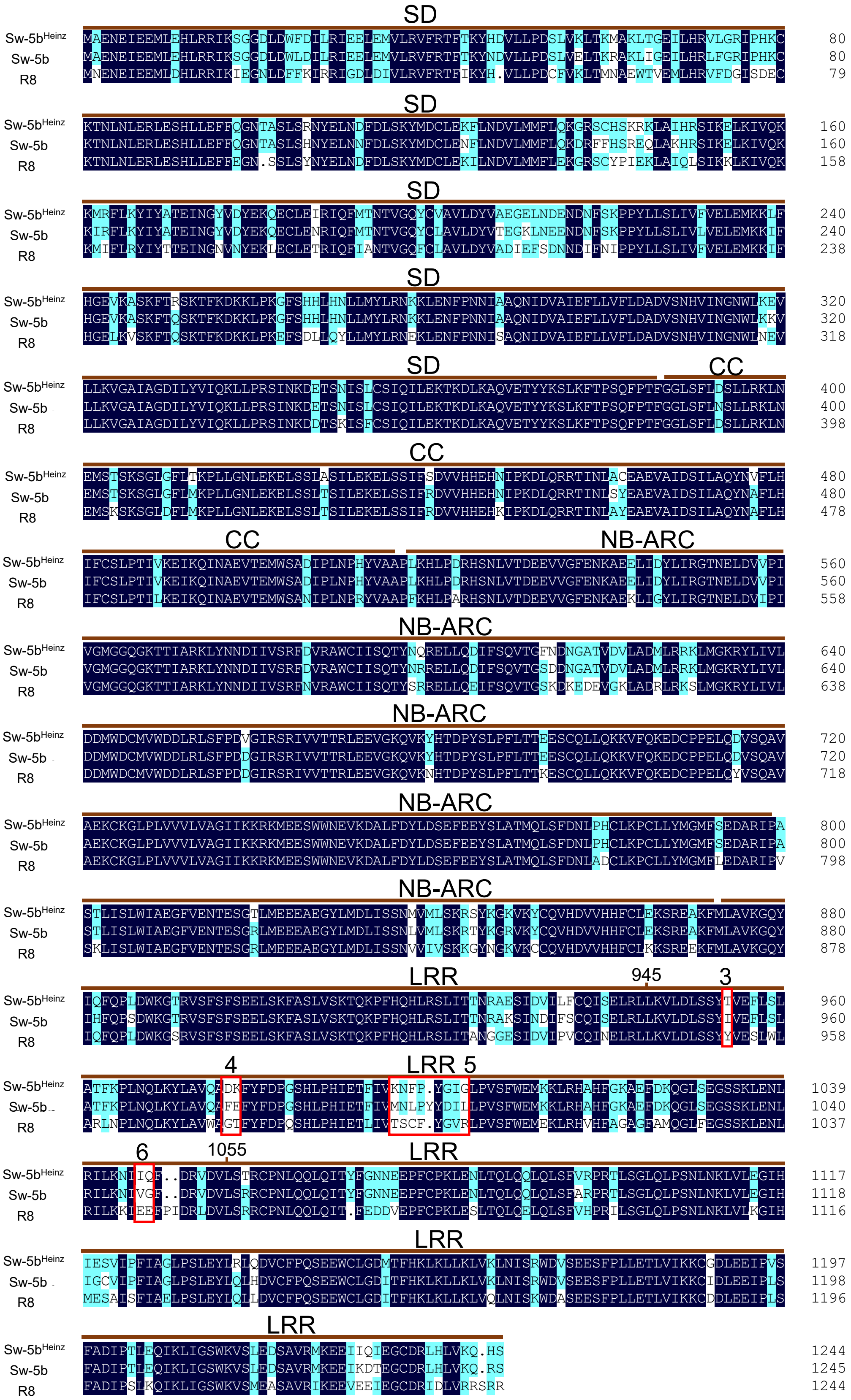

Appendix Figure S9. Amino acid sequence alignment of Sw-5b^{Heinz}, Sw-5b and R8. The domains of SD, CC, NB-ARC and LRR are shown upper the aligned sequence. The 945-1055 amino acid region encompassing polymorphic sites 3-6 is indicated with red box.

Appendix Table S1. List of primers used in this study

Plasmid constructions	Primer	Sequence (5'-3')	Purpose
p2300S-YFP-Sw-5b	PV953 (F)	GG***GGTACC***ATGGTGAGCAAGGGCGAGG ***Kpn I***	To amplify YFP-tagged Sw-5b and cloned into p2300S
	PV954(R)	AGATCTGTACAGCTCGTCCA	
	PV955(F)	TGGACGAGCTGTACAGATCTATGGCTGAAAATGAAATTGAG	
	PV312 (R)	AA***CTGCAG***TCAATCTGAGCGTTGTTTGACGAGG ***Pst I***	
p2300S-YFP-Sw-5b^{Heinz}	PV953 (F)	GG***GGTACC***ATGGTGAGCAAGGGCGAGG ***KpnI***	To amplify Sw-5b^{Heinz} and YFP, respectively, then the Sw-5b^{Heinz} was fused in frame with YFP at the N-terminus and cloned into p2300S
	PV954 (R)	AGATCTGTACAGCTCGTCCA	
	PV955 (F)	TGGACGAGCTGTACAGATCTATGGCTGAAAATGAAATTGAG	
	PV312 (R)	AA***CTGCAG***TCAATCTGAGCGTTGTTTGACGAGG ***Pst I***	
p2300S-FLAG-Sw-5b NB-LRR	P1474 (F)	CG***GGTACC***atgGATTACAAGGATGATGATGATAAGccattaaaacatctgccg gatcga ***Kpn I***	To amplify FLAG-tagged Sw-5b NB-LRR and cloned into p2300S
	PV312 (R)	AA***CTGCAG***TCAATCTGAGCGTTGTTTGACGAGG ***Pst I***	
pGBKT7-Sw-5b SD	Wcl259 (F)	CATG***CCATGG***AGATGGCTGAAAATGAAATTG ***Nco I***	To amplify Sw-5b SD and cloned into pGBKT7
	Wcl260 (R)	AA***CTGCAG***TCAGGGGAACTGAGATGGAGT ***Pst I***	
pGBKT7-Sw-5b NB	Wcl261 (F)	CATG***CCATGG***AGCCATTAAAACATCTGCCGGATC ***Nco I***	To amplify Sw-5b NB and cloned into pGBKT7
	Wcl262 (R)	AA***CTGCAG***TCATGTTGTGAGGAATGGAAG ***Pst I***	
pGADT7-NbSBP1	Wcl275 (F)	CG***GAATTC***ATGGCTCTTCCTCATCACCATC ***EcoR I***	To amplify SBP1 and cloned into pGADT7
	Wcl276 (R)	CG***GGATCC***CTATATGTAAACTTCCATGCC ***BamH I***	
pNbSBP1-nLUC	Wcl233 (F)	GG***GGTACC***ATGGCTCTTCCTCATC ***Kpn I***	To amplify SBP1 and cloned into p-nLUC
	Wcl345 (R)	ACGC***GTCGAC***TATGTAAACTTCCATG ***Sal I***	
pcLUC-Sw-5b SD	Wcl346 (F)	GG***GGTACC***ATGGCTGAAAATGAAATTG ***Kpn I***	To amplify Sw-5b SD and cloned into p-cLUC
	Wcl347 (R)	CG***GGATCC***TCAGGGGAACTGAGATGGAGT ***BamH I***	
pcLUC-Sw-5b NB	Wcl348 (F)	GG***GGTACC***CCATTAAAACATCTGCCGGATC ***Kpn I***	To amplify Sw-5b NB and cloned into p-cLUC
	Wcl349 (R)	CG***GGATCC***TCATGTTGTGAGGAATGGAAG ***BamH I***	

pNbSBP1^{RM}-nLUC<br>p2300S-NbSBP1^{RM} –RFP/YFP	Wc267 (F)<br>Wcl268 (R)	CTATTGTTACCCTCTAAGCATCTCTGCCTGTCTAAAG<br>CTTTAGACAGGCAGAGATGCTTAGAGGGTAACAATAG	To generate amino acid substitution C to S at position 306 and 312 (C306/312S) in SBP1
p2300S-NbSBP1–RFP	Wcl233 (F)<br>Wcl269 (F)<br>Wcl270 (R)<br>Wcl271 (R)	GG***GGTACC***ATGGCTCTTCCTCATC ***Kpn I***<br>GGCATGGAAGTTTACATAGGATCCATGGTGAGCAAGGGCG<br>CGCCCTTGCTCACCATGGATCCTATGTAAACTTCCATGCC<br>AA***CTGCAG***TTAAGATCTGTACAGCTCG ***Pst I***	To amplify RFP-tagged SBP1 and cloned into p2300S
p2300S-NbSBP1–YFP	Wcl272 (F)<br>Wcl273 (R)<br>Wcl274 (R)	GGCATGGAAGTTTACATAGGATCCATGGTGAGCAAGGGC<br>GCCCTTGCTCACCATGGATCCTATGTAAACTTCCATGCC<br>AA***CTGCAG***TTAAGATCTGTACAGCTCG ***Pst I***	To amplify YFP-tagged SBP1 and cloned into p2300S
p2300S-Sw-5b SD-FLAG	Wcl346 (F)<br>Wcl350 (R)	GG***GGTACC***ATGGCTGAAAATGAAATTG ***Kpn I***<br>AA***CTGCAG***TCACTTATCATCATCATCCTTGTAATCCATGGGGAACTG AGATGGAG ***Pst I***	To amplify FLAG-tagged Sw-5b SD and cloned into p2300S
p2300S-YFP-Sw-5b SD	PV953 (F)<br>PV954(R)<br>PV955(F)<br>Wcl347 (R)	GG***GGTACC***ATGGTGAGCAAGGGCGAGG ***Kpn I***<br>AGATCTGTACAGCTCGTCCA<br>TGGACGAGCTGTACAGATCTATGGCTGAAAATGAAATTGAG<br>CG***GGATCC***TCAGGGGAACTGAGATGGAGT ***BamH I***	To amplify YFP-tagged Sw-5b SD and cloned into p2300S
pGBK01/Cas9-NbSBP1	Wcl583 (F)<br>Wcl584 (R)	TGATTGCTTCGTGAAAAAGAGGCAG<br>AAACCTGCCTCTTTTTCACGAAGCA	To generate sgRNA of *NbSBP1* and cloned into pGBK01/Cas9
p2300S-YFP-R8	PV953 (F)<br>PV954(R)<br>Wcl351 (F)<br>Wcl352 (R)	GG***GGTACC***ATGGTGAGCAAGGGCGAGG ***Kpn I***<br>AGATCTGTACAGCTCGTCCA<br>CATGGACGAGCTGTACACTATGAATGAAAATGAAATTG<br>AA***CTGCAG***TCAATCTCTTCGACTTC ***Pst I***	To amplify YFP-tagged R8 and cloned into p2300S
p2300S-FLAG-Rpi-blb2	Wcl353 (F)<br>Wcl354 (R)	CG***GGTACC***atgGATTACAAGGATGATGATGATAAGATGGAAAAACGA AAAG ***Kpn I***<br>CG***GGATCC***CTACTTAAATAACGGG ***BamH I***	To amplify FLAG-tagged Rpi-blb2 and cloned into p2300S
pBin-FLAG-Sw-5b	HS198 (F)	TGACAAGGGATCCTCTAGAATGGCTGAAAATGAAATTGAG	To amplify *Sw-5b/Sw-5b^{D857V}* and cloned into

	HS199 (R)	TTGCATGCCTGCAGGTCGACTCAATCTGAGCGTTGTTTG	pBinPLUS-FLAG
pTRV2-SlSBP1	Wcl240 (F)<br>Wcl241 (R)	GC***<u>TCTAGA</u>***TGGCGCTTCCTCATCACC ***XbaI***<br>CG***<u>GGATCC</u>***GTTATTCTCCATAAAATCTTG ***BamH I***	To amplify 300 bp of SlSBP1 and cloned into pTRV2
pET28a-His-NbSBP1^{RM}-HA-His	Wcl741 (F)<br>Wcl742 (R1)<br>Wcl743 (R2)	GCAAATGGGTCGCGGATCCATGGCTCTTCCTCATC<br>TAGTCAGGAACATCGTATGGGTAtatgtaaacttccatg<br>GGTGGTGGTGCTCGAGGGCATAGTCAGGAACATCGTATG	To amplify NbSBP1-HA and cloned into pET28a
pGEX-2TK-Sw-5b<br>pGEX-2TK-Sw-5b^{D857V}	Wcl222 (F)<br>Wcl223 (R)	CATCTGTTGGATCCATGGCTGAAAATGAAA<br>GATGAATTCCCGGTCATCAATCTGAGCGTTG	To amplify Sw-5b / Sw-5b^{D857V} and cloned into pGEX-2TK
pGEX-2TK-Sw-5b SD	PV931 (F)<br>P2702 (R)	CG***<u>GGATCC</u>***ATGGCTGAAAATGAAATTGA ***BamH I***<br>CG***<u>GGATCC</u>***TCAGGGGAACTGAGATGGAGTA ***BamH I***	To amplify Sw-5b SD and cloned into pGEX-2TK
pGEX-2TK-Sw-5b NB-LRR	Wcl220 (F)<br>Wcl221 (R)	TCC***<u>CCCGGG</u>***CCATTAAAACATCTG ***Sma I***<br>TCC***<u>CCCGGG</u>***TCAATCTGAGCGTTG ***SmaI***	To amplify Sw-5b NB-ARC-LRR and cloned into pGEX-2TK
p2300S-FLAG-Ub	Wcl227 (F)<br>Wcl228 (R)	GG***<u>GGTACC</u>***ATGGATTACAAGGATGATGATGATAAGATGCAGATCTTC GTGAAAACCC ***Kpn I***<br>AA***<u>CTGCAG</u>***TTACTTGCGGCAGATCATC ***Pst I***	To amplify FLAG-tagged Ub and cloned into p2300S
qRT-Sw-5b<br>qRT-Sw-5b NB-LRR	Wcl463 (F)<br>Wcl464 (R)	CAAAGGACTGCCCCTAGTGG<br>TGCTGGAATTCTTGCGTCCT	To detect the mRNA expression level of Sw-5b and Sw-5b NB-LRR
qRT-SlSBP1	Wcl465 (F)<br>Wcl466 (R)	GCGCTTCCTCATCACCATCT<br>AATCTTGCTCCTTGGGCCTC	To detect the mRNA expression level of SBP1
qRT-Sw-5b SD	Wcl534 (F)<br>Wcl535 (R)	GGTGATCTGGATTGGCTCGA<br>GGCCCTCTTTGTGAGTTCGA	To detect the mRNA expression level of Sw-5b SD
qRT-Sw-5b^{Heinz}	Wcl729 (F)<br>Wcl730 (R)	GAATCTATTGATGTGATTCTCTTC<br>CCCTATACCATAAGGAAAATTCT	To detect the mRNA expression level of Sw-5b^{Heinz}
qRT-R8	Wcl847 (F)<br>Wcl848 (R)	ATCTGCCAGCTCGACATAGC<br>TTGTCCTTGGAGCCCGTAAC	To detect the mRNA expression level of R8

qRT-Rpi-blb2	Wcl854 (F)<br>Wcl855 (R)	GCAATTGGGCTTCCTCCTCT<br>CATCAGCCTGATCCTCCCA	To detect the mRNA expression level of Rpi-blb2
p2300s-YFP-SD^{Heinz}-CNL$^{Sw\text{-}5b/D857V}$	P3882 (F)<br>P3517 (R)<br>P3518 (F)<br>P3883 (R)	ACGAGCTGTACAGATCTTAAATGGCTGAAAATGAAATTGA<br>CAGAAAGCTCAATCCACCAAAGGTGGGGAACTGAGATGGAGTAAATTT<br>AAATTTACTCCATCTCAGTTCCCCACCTTTGGTGGATTGAGCTTTCTG<br>TCAATTTCATTTTCAGCCATTTAAGATCTGTACAGCTCGT	To amplify Heinz SD and linearized plasmid 2300S, respectively, then the Heinz SD was fused in the middle of YFP and Sw-5b CC-NB-ARC-LRR, finally generated YFP-tagged chimeric Sw-5b mutant
p2300S-YFP-Sw-5b$^{LRRM3\text{-}6}$<br>p2300S-YFP-Sw-5b$^{D857V/LRRM3\text{-}6}$	P3648 (F)<br>P3624 (R)<br>P3625 (F)<br>P3649 (R)	CCATTAAAACATCTGCCGGATCGACATAGCAATCTTGTAACTGATGAG<br>TCAATCTGAGCGTTGTTTGACGAGGTGTAAACGATCACATCCTTCAGT<br>ACTGAAGGATGTGATCGTTTACACCTCGTCAAACAACGCTCAGATTGA<br>CTCATCAGTTACAAGATTGCTATGTCGATCCGGCAGATGTTTTAATGG	To amplify Sw-5b NB-ARC-LRR$^{M3\text{-}6}$ and linearized plasmid 2300S, respectively, then the Sw-5b NB-ARC-LRR$^{M3\text{-}6}$ was fused in frame with YFP-Sw-5b SD-CC at the C-terminus, finally generated YFP-tagged chimeric Sw-5b mutants
p2300S-YFP-(SD-ARC)$^{Sw\text{-}5bD857V}$-LRR^{Heinz}	PV953 (F)<br>Wcl357 (R)<br>Wcl358 (F)<br>Wcl359 (R)	GG***GGTACC***ATGGTGAGCAAGGGCGAGG ***Kpn I***<br>CCCTTCACTGCAAGCATAAACTTTGCTTCTC<br>GTAGAGAAGCAAAGTTTATGCTTGCAGTGAAGGG<br>AA***CTGCAG***TCAATCTGAGTGTTGTTTG ***Pst I***	To amplify (YFP-SD-ARC)$^{Sw\text{-}5bD857V}$ and LRR^{Heinz}, respectively, then overlap and cloned into p2300S
p2300S-YFP-SD^{Heinz}-(NB-ARC)$^{Sw\text{-}5b/D857V}$-LRR$^{M3\text{-}6}$	W1260 (F1)<br>W1261 (R1)<br>W1262 (F2)<br>W1263 (R2)	CGAGCTCGGTACCATGGTGAGCAAGGG<br>AAACTTTGCTTCTCTAC<br>AAACTTTGCTTCTCTAC<br>CCCTTGCTCACCATGGTACCGAGCTCG	To amplify YFP-SD^{Heinz}-(NB-ARC)$^{Sw\text{-}5b}$ and linearized plasmid 2300S, respectively, then the YFP-SD^{Heinz}-(NB-ARC)$^{Sw\text{-}5b}$ was fused fused in frame with LRR$^{M3\text{-}6}$ at the N-terminus, finally generated YFP-tagged chimeric Sw-5b mutant

pET28a-TZSV NSm-2Myc	W1265 (F)<br>W1277 (R1)<br><br>W1278 (R2)	GGACAGCAAATGGGTCGCATGTCTCGCATTACTAAC<br>CTCCTCCAGGTCCTCCTCTGAGATCAGCTTCTGCTCGAAATCTAATGTGTTG<br>GGTGGTGGTGGTGGTGCAGGTCCTCCTCTGAGATCAGCTTCTGCTCCTCCAGGTCCTCC	To amplify TZSV NSm-2Myc and cloned into pET28a
pET28a-TSWV NSm-2Myc	W1279 (F)<br>W1280 (R1)	GGACAGCAAATGGGTCGCATGTTGACTTTTTTTG<br>GCTCCTCCAGGTCCTCCTCTGAGATCAGCTTCTGCTCTATCTCATCAAAAGATAAC	To amplify TSWV NSm-2Myc and cloned into pET28a
pBin-YFP-SD$^{K79/81/153R}$	W1269 (F1)<br>W1270 (R1)<br>W1271 (F2)<br>W1272 (R2)	GATTCCACATCGGTGTCGGACTAACCTTAATC<br>CAATTTTCAGTTCCCGTATTGATC<br>GATCAATACGGGAACTGAAAATTG<br>GATTAAGGTTAGTCCGACACCGATGTGGAATC	To amplify SD$^{K79/81/153R}$ and linearized plasmid pBin, respectively, then the SD$^{K79/81/153R}$ was fused in frame with YFP at the C-terminus, finally generated YFP-tagged chimeric SD mutant
pBin-Sw-5b 945-1055-YFP	W551 (F)<br>W552 (R)	CGAGCTCGGTACCATGAAAGTCTTGGATTTG<br>CCTTGCTCACCATCCCTAACACATCCACCC	To amplify Sw-5b 945-1055 and cloned into pBinPLUS-YFP
pBin-Sw-5b 945-1055-YFP K946/964/970R	W1293 (F1)<br><br>W1294 (F2)<br><br>W552 (R)	CTTTCGCGAGCTCGGTACCATGCTTCGGGTCTTGGATTTGAGTTCTTATATTGTGGAGT<br>CTTATATTGTGGAGTTTTTGTCGTTAGCTACATTCCGGCCACTAAATCAGCTGCGGTAC<br>CCTTGCTCACCATCCCTAACACATCCACCC	To amplify Sw-5b 945-1055 K946/964/970R and cloned into pBinPLUS-YFP
pBin-Sw-5b 945-1055-YFP K1013/1014R	W551 (F1)<br>W1276 (R1)<br>W1298 (F2)<br>W552 (R2)	CGAGCTCGGTACCATGAAAGTCTTGGATTTG<br>CCTTAACCGCCGCATTTCCCAAAAAG<br>GGGAAATGCGGCGGTTAAGGC<br>CCTTGCTCACCATCCCTAACACATCCACCC	To amplify Sw-5b 945-1015 and Sw-5b 1012-1055, respectively, then overlap and cloned into pBinPLUS-YFP
pBin-Sw-5b 945-1055-YFP K1022/1027R	W551 (F1)<br>W1296 (R1)	CGAGCTCGGTACCATGAAAGTCTTGGATTTG<br>CTGCCGGTCAAATTCAGCCCGACC	To amplify Sw-5b 945-1028 and Sw-5b 1020-1055, respectively, then overlap and

	W1297 (F2)	GGTCGGGCTGAATTTGACCGGCAG	cloned into pBinPLUS-YFP
	W552 (R2)	CCTTGCTCACCATCCCTAACACATCCACCC	
pBin-Sw-5b 945-1055-YFP K1036/1044R	W551 (F)	CGAGCTCGGTACCATGAAAGTCTTGGATTTG	
	W1295 (R1)	AACACATCCACCCTATCAAATCCAACAATATTCCTTAATATCCTCAAATTTTCCAACCT	To amplify Sw-5b 945-1055 K1036/1044R and cloned into pBinPLUS-YFP
	W552 (R2)	CCTTGCTCACCATCCCTAACACATCCACCC	
