# Supplementary material for: A viral effector blocks the turnover of a plant NLR receptor to trigger a robust immune response
Source: EMBO J. 2024 Jul 17;43(17):7. doi: 10.1038/s44318-024-00174-6 (PMC11377725; doi:10.1038/s44318-024-00174-6)
Supplement: Supplementary file 10 — Expanded View Figures [file 44318_2024_174_MOESM10_ESM.pdf]

Expanded View Figures

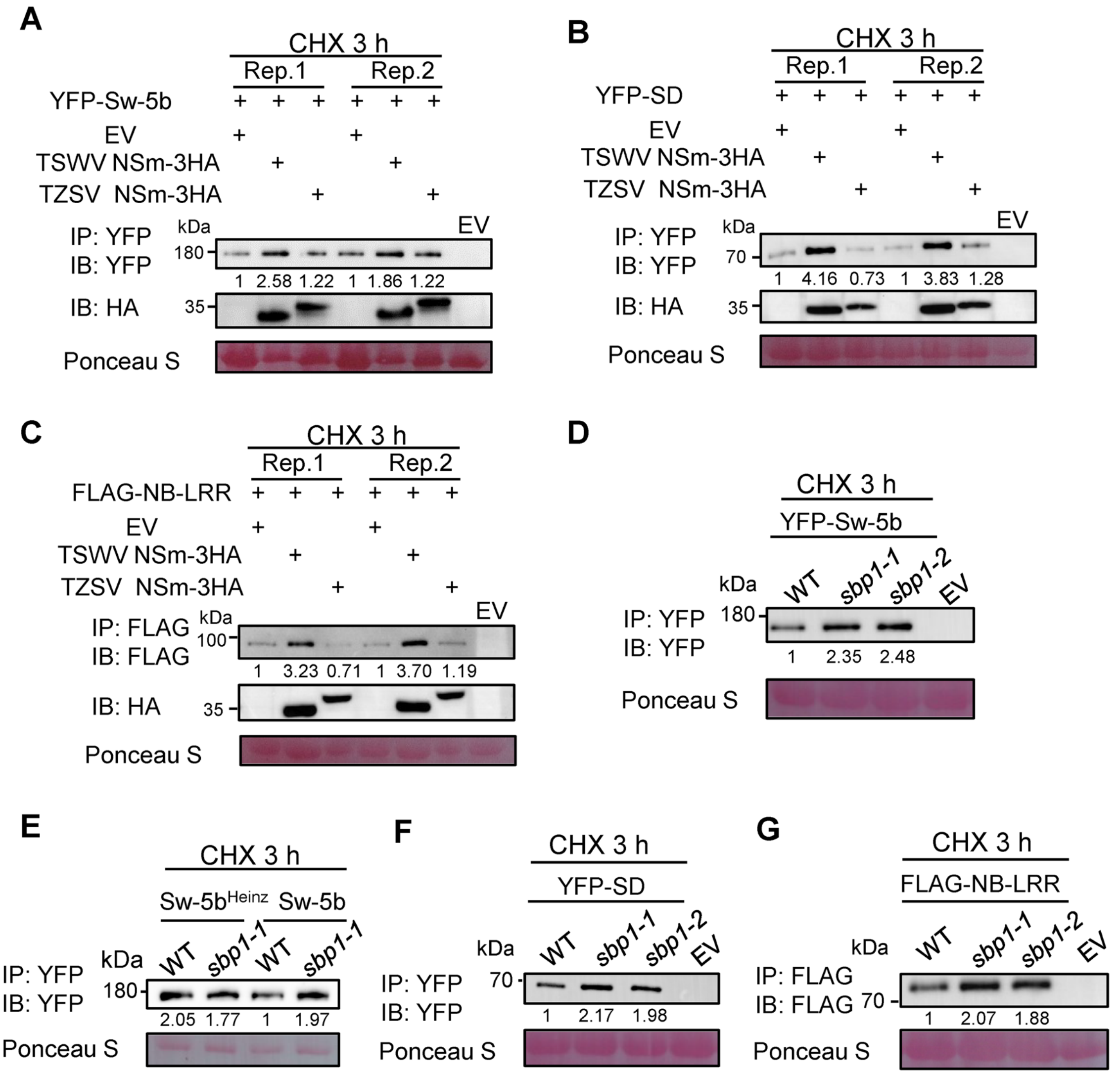

Figure EV1. The TSWV NSm protects Sw-5b NLR from degradation by the plant 26S proteasome.

(**A–C**) The accumulation of Sw-5b, SD and NB-LRR in the presence of TSWV NSm or TZSV NSm in *N. benthamiana* leaves. YFP-Sw-5b (**A**), YFP-SD (**B**) or FLAG-NB-LRR (**C**) was co-expressed with the pCambia2300 empty vector (EV), TSWV NSm, or TZSV NSm for 22 h. Samples were treated with 10 μg/mL cycloheximide (CHX) at 19 hpi to block protein synthesis. Protein accumulation was detected at 3 h post CHX treatment by immunoblot using YFP, FLAG, and HA-specific antibodies. (**D–G**) Protein accumulation analysis of YFP-Sw-5b (**D**), YFP-Sw-5b^{Heinz} (**E**), YFP-SD (**F**), and FLAG-NB-LRR (**G**) in wild-type (WT) and *SBP1* knockout *N. benthamiana* plants. YFP-Sw-5b, YFP-Sw-5b^{Heinz}, YFP-SD and FLAG-NB-LRR were expressed in WT, *sbp1-1* or *sbp1-2* mutant *N. benthamiana* leaves and treated with 10 μg/mL CHX at 21 hpi. Total protein was extracted from the samples 24 hpi and analyzed for protein accumulation by immunoblot using YFP and FLAG-specific antibody. Protein accumulation levels were quantified by ImageJ software. Source data are provided as a Source Data file. All experiments were repeated at least three times with similar results. Source data are available online for this figure.

 © The Author(s)

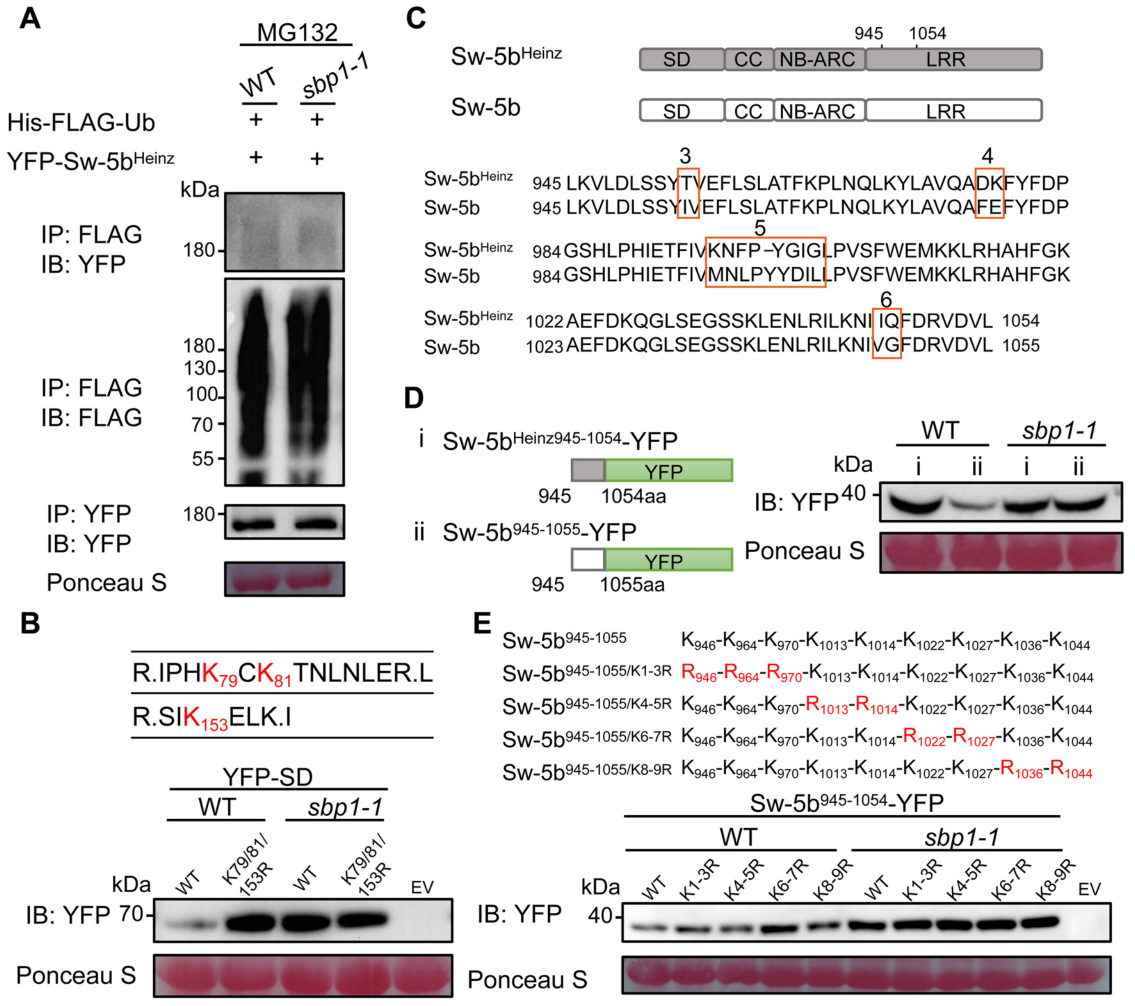

Figure EV2. Determination of key amino acids within Sw-5b SD and LRR3-6 that were ubiquitinated by SBP1.

(**A**) In vivo ubiquitination assay to analyze the ubiquitination of Sw-5b^{Heinz} in WT and *SBP1* knockout *N. benthamiana* plants. YFP-Sw-5b^{Heinz} was co-expressed with FLAG-Ub in WT or *sbp1-1* mutant *N. benthamiana* leaves, 25 μM MG132 was infiltrated into the leaves at 16 hpi. The ubiquitination of Sw-5b^{Heinz} was detected at 24 hpi by immunoprecipitation using anti-FLAG beads followed by detection with YFP-specific antibodies. The overall ubiquitinated proteins were detected using FLAG-specific antibodies. (**B**) Determination of amino acids within Sw-5b SD that was ubiquitinated by SBP1. The ubiquitinated lysine residues K79, K81, and K153 identified by LC–MS/MS are shown in red (upper panel). The protein accumulation of wild-type SD and SD$^{K79/81/153R}$ mutant was analyzed in wild-type and *sbp1-1* mutant *N. benthamiana* plants (lower panel). (**C–E**) Determination of amino acids within Sw-5b LRR3-6 that was ubiquitinated by SBP1. The schematic diagram of Sw-5b and Sw-5b^{Heinz} homolog showing the SD, CC, NB-ARC and LRR domains is shown in the top panel (**C**). The polymorphic, 945–1054 amino acid (aa) region encompassing polymorphic sites 3–6 of the LRR domain (LRR3-6) of the Sw-5b and the Sw-5b^{Heinz} is shown in the bottom panel (**C**). The key amino acids for NSm recognition is shown in the red boxed region. The 945–1054 aa region of Sw-5b^{Heinz} or the 945–1055 aa region of Sw-5b encompassing LRR3-6 was fused to the N-terminus of YFP. The accumulation levels of Sw-5b$^{Heinz945\text{-}1054}$-YFP and Sw-5b$^{945\text{-}1055}$-YFP were analyzed in WT or *sbp1-1* mutant leaves at 24 hpi by immunoblot using YFP-specific antibodies (**D**). All lysine sites in Sw-5b LRR3-6 were analyzed by mutating K946-K964-K970, K1013-K1014, K1022-K1027, K1036-K1044 to R in four mutants. Arginine used to replace lysine sites in Sw-5b$^{945\text{-}1055}$ mutants are shown in red. The protein accumulation levels of Sw-5b$^{945\text{-}1055}$-YFP and its mutants were analyzed in WT and *sbp1-1* mutant *N. benthamiana* plants using YFP-specific antibodies (**E**). Source data are provided as a Source data file. All experiments were repeated at least three times with similar results. Source data are available online for this figure.

© The Author(s)

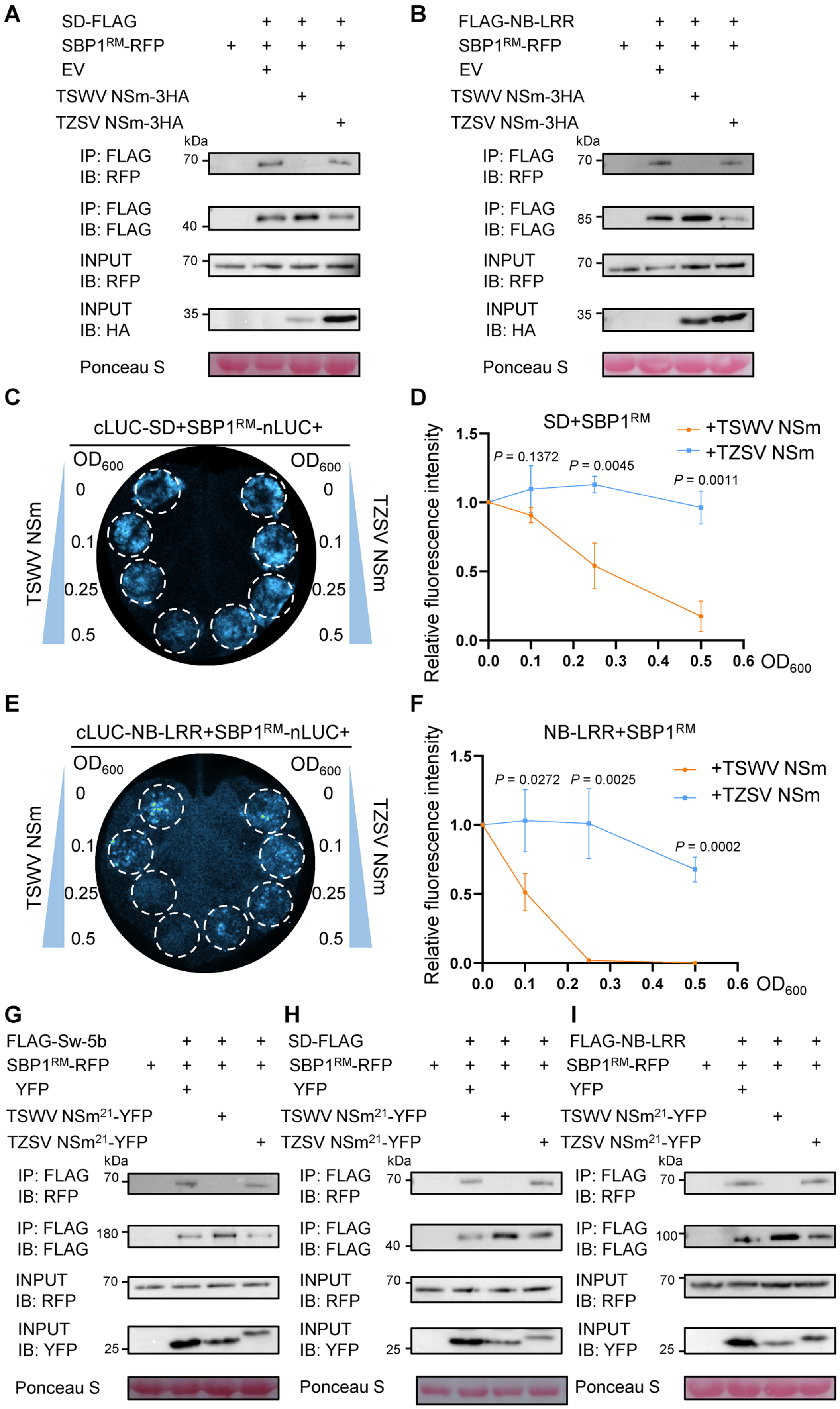
A
SD-FLAG
SBP1RM-RFP
EV
TSWV NSm-3HA
TZSV NSm-3HA
IP: FLAG IB: RFP
IP: FLAG IB: FLAG
INPUT IB: RFP
INPUT IB: HA
Ponceau S
B
FLAG-NB-LRR
SBP1RM-RFP
EV
TSWV NSm-3HA
TZSV NSm-3HA
C
cLUC-SD+SBP1RM-nLUC+
OD600
TSWV NSm
TZSV NSm
D
SD+SBP1RM
+TSWV NSm
+TZSV NSm
P = 0.1372
P = 0.0045
P = 0.0011
Relative fluorescence intensity
OD600
E
cLUC-NB-LRR+SBP1RM-nLUC+
F
NB-LRR+SBP1RM
P = 0.0272
P = 0.0025
P = 0.0002
G
FLAG-Sw-5b
SBP1RM-RFP
YFP
TSWV NSm21-YFP
TZSV NSm21-YFP
IB: YFP
H
SD-FLAG
I
FLAG-NB-LRR

Figure EV3. Tospoviral NSm and conserved NSm^{21} peptide region interferes with the interaction between Sw-5b and SBP1.

(**A**, **B**) TSWV NSm interferes with the interactions of Sw-5b SD and NB-LRR with SBP1. SD-FLAG (**A**) or FLAG-NB-LRR (**B**) was immunoprecipitated with SBP1^{RM}-RFP in the presence of EV, TSWV NSm, or TZSV NSm, with SBP1^{RM}-RFP alone as a control. The co-IP assay was carried out at 21 hpi and immunoblots were probed using YFP, FLAG, RFP, and HA-specific antibodies. (**C**, **E**) Split-luciferase complementation assay showing the inhibitory effects of TSWV NSm on the interactions of Sw-5b SD (**C**) and NB-LRR (**E**) with SBP1. The cLUC-SD or cLUC-NB-LRR was co-expressed with SBP1^{RM}-nLUC in the presence of increasing amounts of Agrobacteria carrying TSWV NSm or TZSV NSm. Luciferase activity was detected at 36 hpi. (**D**, **F**) The fluorescence intensity of the interaction between SD (**D**) and NB-LRR (**F**) with SBP1^{RM} from (**C**) and (**E**) was quantified using ImageJ. Data are presented as means ± SD ($n = 3$ biologically independent samples). Data were analyzed by two-sided Student's *t*-test. The exact *P* values are indicated in the graphs. (**G–I**) TSWV NSm^{21}-YFP interferes with the interaction of Sw-5b, SD, and NB-LRR with SBP1. YFP-Sw-5b (**G**), SD-FLAG (**H**), or FLAG-NB-LRR (**I**) was immunoprecipitated with SBP1^{RM}-RFP in the presence of EV, TSWV NSm^{21}-YFP, or TZSV NSm^{21}-YFP. The co-IP assay was carried out at 21 hpi and immunoblots were probed using YFP, RFP, and FLAG-specific antibodies. The size of protein was shown on the left. IB, immunoblot with specific antibody; IP, immunoprecipitation with specific antibody. Ponceau S staining was used to show the amount of protein loaded. Experiments were repeated at least three times with similar results. Source data are provided as a Source data file. Source data are available online for this figure.

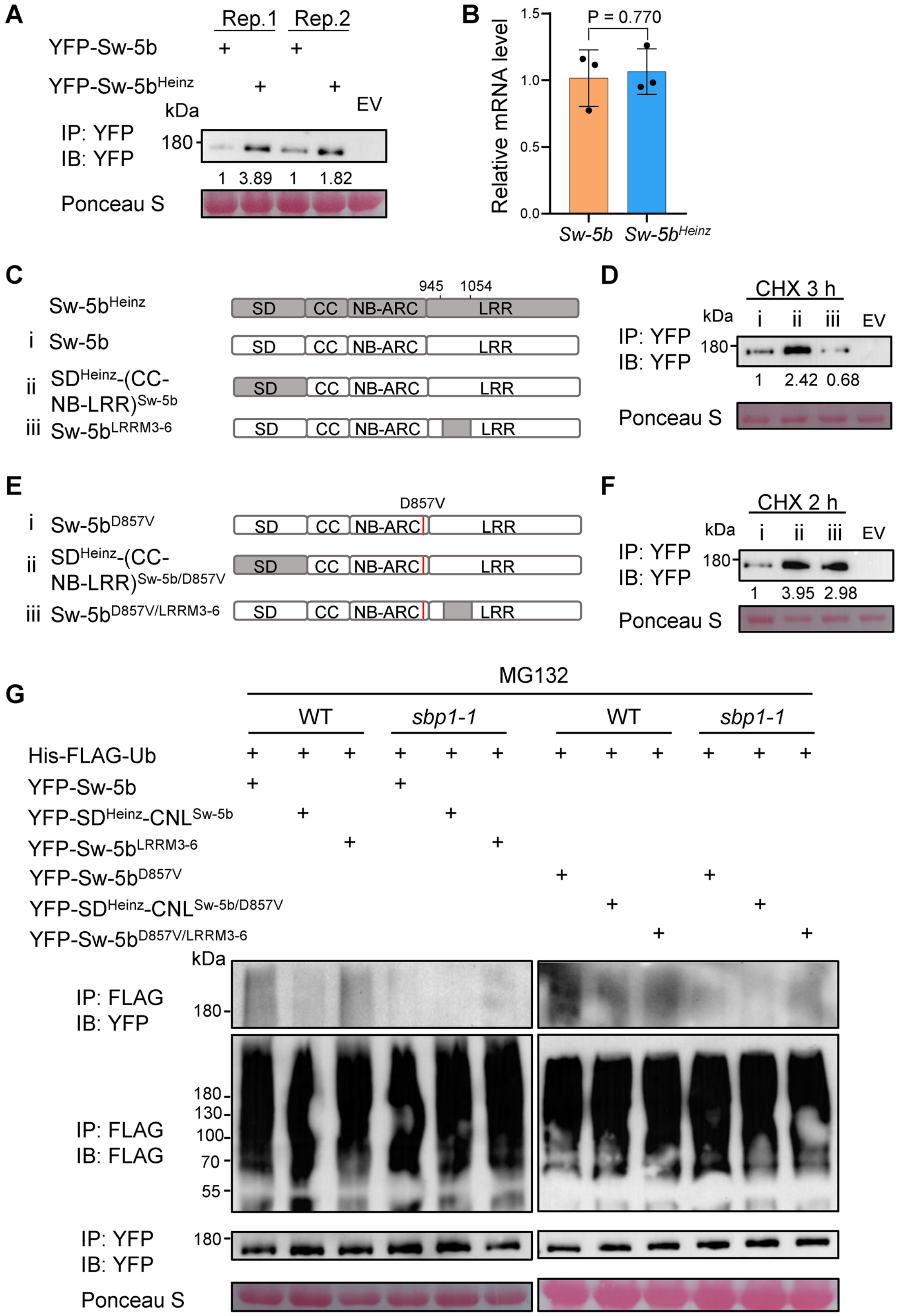
A
Rep.1 Rep.2
YFP-Sw-5b + +
YFP-Sw-5bHeinz + +
EV
kDa
IP: YFP
IB: YFP
180
1 3.89 1 1.82
Ponceau S
B
P = 0.770
Relative mRNA level
1.5
1.0
0.5
0.0
Sw-5b
Sw-5bHeinz
C
945 1054
Sw-5bHeinz SD CC NB-ARC LRR
i Sw-5b SD CC NB-ARC LRR
ii SDHeinz-(CC-NB-LRR)Sw-5b SD CC NB-ARC LRR
iii Sw-5bLRRM3-6 SD CC NB-ARC LRR
D
CHX 3 h
kDa i ii iii EV
IP: YFP
IB: YFP
180
1 2.42 0.68
Ponceau S
E
D857V
i Sw-5bD857V SD CC NB-ARC LRR
ii SDHeinz-(CC-NB-LRR)Sw-5b/D857V SD CC NB-ARC LRR
iii Sw-5bD857V/LRRM3-6 SD CC NB-ARC LRR
F
CHX 2 h
kDa i ii iii EV
IP: YFP
IB: YFP
180
1 3.95 2.98
Ponceau S
G
MG132
WT sbp1-1 WT sbp1-1
His-FLAG-Ub
YFP-Sw-5b
YFP-SDHeinz-CNLSw-5b
YFP-Sw-5bLRRM3-6
YFP-Sw-5bD857V
YFP-SDHeinz-CNLSw-5b/D857V
YFP-Sw-5bD857V/LRRM3-6
kDa
IP: FLAG
IB: YFP
180
IP: FLAG
IB: FLAG
180
130
100
70
55
IP: YFP
IB: YFP
180
Ponceau S

 © The Author(s)

Figure EV4. Protein accumulation and ubiquitination analysis of chimeric Sw-5b variants.

(**A**) The accumulation of Sw-5b and Sw-5b^{Heinz} in *N. benthamiana* leaves. YFP-Sw-5b was expressed in one-half leaf of *N. benthamiana* plant and YFP-Sw-5b^{Heinz} expressed in another half of the same leaf. Protein accumulation was detected by immunoblot at 24 hpi using YFP-specific antibody. (**B**) The relative transcript expression levels of YFP-Sw-5b and YFP-Sw-5b^{Heinz}. Total RNA was extracted at 24 hpi for qRT-PCR analysis. Data are presented as means ± SD ($n = 3$ biologically independent samples). Data were analyzed by two-sided Student's *t*-test. The exact *P* values are indicated in the graph. (**C**) The schematic diagram showing the architecture of the chimeric proteins used to assay the role of SD and LRR (945–1054 aa) in maintaining the homeostasis of the inactive Sw-5b. (**D**) The accumulation of YFP-Sw-5b, YFP-SD^{Heinz}-(CC-NB-LRR)$^{Sw\text{-}5b}$, and YFP-Sw-5b$^{LRRM3\text{-}6}$ in *N. benthamiana* leaves. The leaves were treated with 10 μg/mL CHX at 21 hpi and protein levels were analyzed by immunoblot 3 h post CHX treatment. (**E**) The schematic diagram showing the architecture of the chimeric proteins used to assay the role of SD and LRR (945–1054 aa) in maintaining the homeostasis of the constitutively active Sw-5b (Sw-5b^{D857V}). (**F**) The accumulation of YFP-Sw-5b^{D857V}, YFP-SD^{Heinz}-(CC-NB-LRR)$^{Sw\text{-}5bD857V}$, and YFP-Sw-5b$^{D857V/LRRM3\text{-}6}$ in *N. benthamiana* leaves at 21 hpi. The leaves were treated with 10 μg/mL CHX at 19 hpi and protein levels were analyzed by immunoblot 3 h post CHX treatment. In (**A**), (**D**), and (**F**), IB, immunoblot with specific antibody; IP, immunoprecipitation with specific antibody. Ponceau S staining was used to show the amount of protein loaded. Protein accumulation level was quantified by ImageJ software. (**G**) In vivo ubiquitination assay of Sw-5b and chimeric Sw-5b variants in WT and *SBP1* knockout *N. benthamiana* plants. YFP-Sw-5b or chimeric Sw-5b variants was co-expressed with FLAG-Ub in leaves of WT or *sbp1-1* mutant *N. benthamiana* leaves, 25 μM MG132 was infiltrated at 16 hpi. The ubiquitination of Sw-5b was detected at 24 hpi by immunoprecipitation using anti-FLAG beads followed by detection with YFP-specific antibodies. The overall ubiquitinated proteins were detected using FLAG-specific antibodies. Experiments were repeated at least three times with similar results. Source data are provided as a Source data file. Source data are available online for this figure.

© The Author(s)

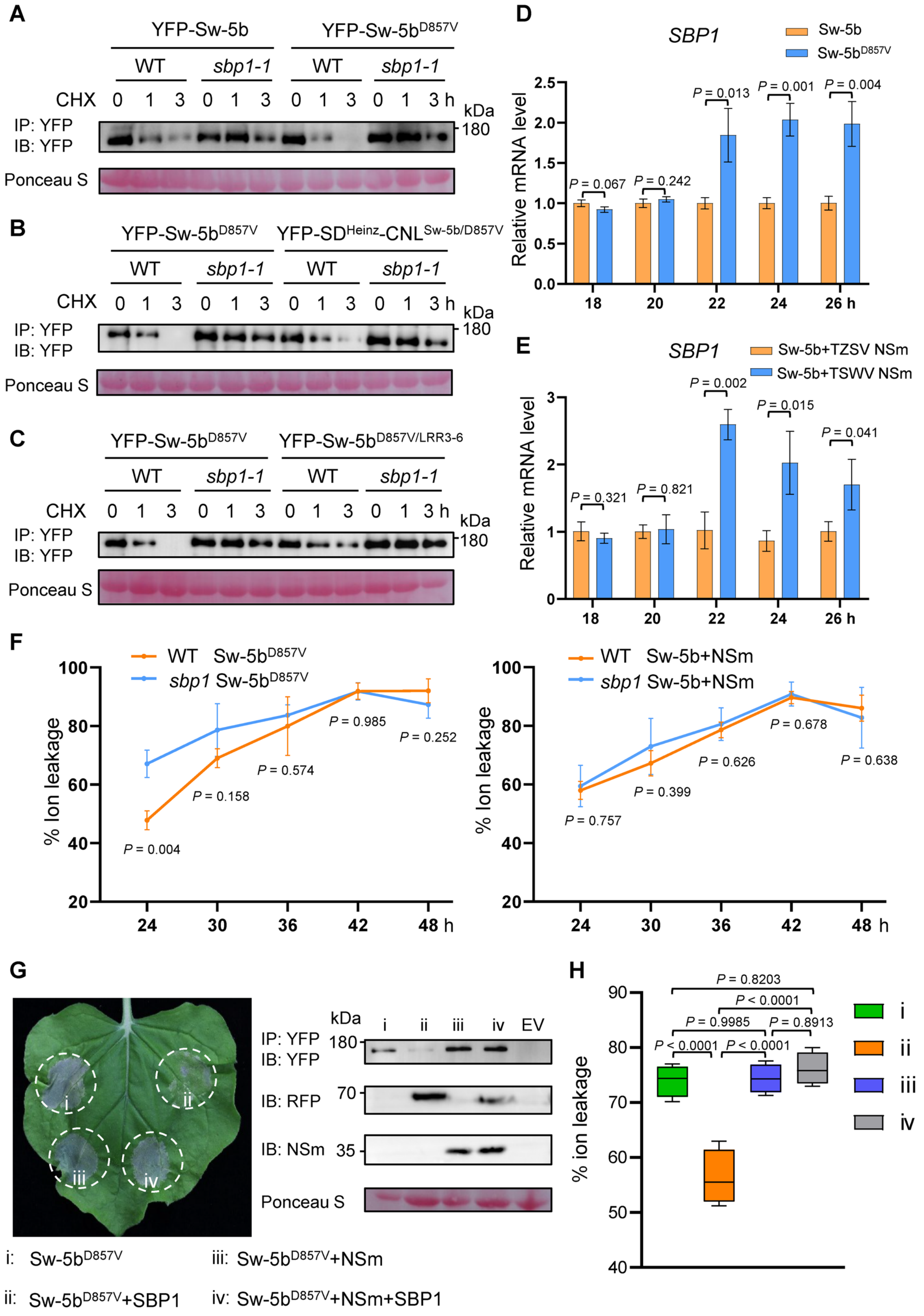
A
YFP-Sw-5b
YFP-Sw-5bD857V
WT sbp1-1 WT sbp1-1
CHX 0 1 3 0 1 3 0 1 3 0 1 3 h
IP: YFP
IB: YFP
kDa
180
Ponceau S
B
YFP-Sw-5bD857V
YFP-SDHeinz-CNLSw-5b/D857V
WT sbp1-1 WT sbp1-1
CHX 0 1 3 0 1 3 0 1 3 0 1 3 h
IP: YFP
IB: YFP
kDa
180
Ponceau S
C
YFP-Sw-5bD857V
YFP-Sw-5bD857V/LRR3-6
WT sbp1-1 WT sbp1-1
CHX 0 1 3 0 1 3 0 1 3 0 1 3 h
IP: YFP
IB: YFP
kDa
180
Ponceau S
D
SBP1
Sw-5b
Sw-5bD857V
Relative mRNA level
P = 0.067
P = 0.242
P = 0.013
P = 0.001
P = 0.004
18 20 22 24 26 h
E
SBP1
Sw-5b+TZSV NSm
Sw-5b+TSWV NSm
Relative mRNA level
P = 0.321
P = 0.821
P = 0.002
P = 0.015
P = 0.041
18 20 22 24 26 h
F
WT Sw-5bD857V
sbp1 Sw-5bD857V
% Ion leakage
P = 0.004
P = 0.158
P = 0.574
P = 0.985
P = 0.252
24 30 36 42 48 h
WT Sw-5b+NSm
sbp1 Sw-5b+NSm
% Ion leakage
P = 0.757
P = 0.399
P = 0.626
P = 0.678
P = 0.638
24 30 36 42 48 h
G
i ii iii iv
kDa i ii iii iv EV
IP: YFP
IB: YFP 180
IB: RFP 70
IB: NSm 35
Ponceau S
i: Sw-5bD857V
ii: Sw-5bD857V+SBP1
iii: Sw-5bD857V+NSm
iv: Sw-5bD857V+NSm+SBP1
H
% ion leakage
P = 0.8203
P < 0.0001
P = 0.9985
P = 0.8913
P < 0.0001
P < 0.0001
i
ii
iii
iv

 © The Author(s)

Figure EV5. Homeostasis of active state Sw-5b is regulated by the E3 ligase SBP1 and the TSWV effector NSm.

(**A**) Protein turnover rate of Sw-5b and Sw-5b^{D857V} in leaves of WT or *sbp1-1* mutant *N. benthamiana* plants. YFP-Sw-5b or YFP-Sw-5b^{D857V} was expressed in *N. benthamiana* leaves and treated with 10 μg/mL CHX at 20 hpi to block protein synthesis. Samples were taken at 0–3 h post CHX treatment. The samples were analyzed by immunoblot using YFP-specific antibodies. (**B**) Protein turnover rate of Sw-5b^{D857V} and SD^{Heinz}-CNL$^{Sw\text{-}5b/D857V}$ in leaves of WT or *sbp1-1* mutant *N. benthamiana* plants. The protein accumulation was examined as described in (**A**). (**C**) Protein turnover rate of Sw-5b^{D857V} and Sw-5b$^{D857V/LRR3\text{-}6}$ in leaves of WT or *sbp1-1* of *N. benthamiana* plants. The protein accumulation was examined as described in (**A**). (**D**) The time course analysis of relative mRNA expression level of *SBP1* in *N. benthamiana* leaves expressing Sw-5b or Sw-5b^{D857V}. (**E**) The time course analysis of relative mRNA expression levels of *SBP1* in *N. benthamiana* leaves co-expressing Sw-5b and elicitor TSWV NSm or non-elicitor TZSV NSm. (**F**) Ion leakage analysis of leaves of WT and *sbp1-1 N. benthamiana* plants co-expressing Sw-5b and NSm or expressing Sw-5b^{D857V} at 6-h intervals from 24 to 48 h post agroinfiltration. NSm here refers to the elicitor TSWV NSm. For (**D**), (**E**), and (**F**), data are presented as means ± SD ($n = 3$ biologically independent samples). The exact *P* values are indicated in the graphs. Data were analyzed by two-sided Student's *t*-test. (**G**, **H**) HR cell death and ion leakage analysis of YFP-Sw-5b^{D857V} and YFP-Sw-5b^{D857V} + NSm in the absence or the presence of SBP1-RFP in *N. benthamiana* leaves. NSm refers to the elicitor TSWV NSm. HR phenotype in the infiltrated leaves was photographed at 4 days post inoculation (**G**). Protein accumulation in (**G**) was detected by immunoblotting using YFP, RFP, and NSm-specific antibodies. The ion leakage was measured at 2 dpi after agroinfiltration and shown in (**H**). Data are shown as the box plots with the interquartile range as the upper and lower confines, minima and maxima as whiskers, and the median as a solid line ($n = 4$); The exact *P* values are indicated in the graph (one-way ANOVA). Source data are provided as a Source data file. All experiments were repeated at least three times with similar results. Source data are available online for this figure.

© The Author(s)
